# Draft Genome Sequences of Lysostaphin-Resistant (K07-204) and Lysostaphin-Susceptible (K07-561) Staphylococcus aureus Sequence Type 72 Strains Isolated from Patients in South Korea

**DOI:** 10.1128/MRA.01057-20

**Published:** 2020-12-03

**Authors:** Nayab Batool, Kwan Soo Ko, Akhilesh Kumar Chaurasia, Kyeong Kyu Kim

**Affiliations:** a Department of Precision Medicine, School of Medicine, Sungkyunkwan University, Suwon, South Korea; b Department of Microbiology, School of Medicine, Sungkyunkwan University, Suwon, South Korea; c Institute of Antimicrobial Resistance and Therapeutics, Sungkyunkwan University, Suwon, South Korea; d Samsung Advanced Institute for Health Sciences and Technology, Samsung Medical Center, Sungkyunkwan University School of Medicine, Seoul, South Korea; Portland State University

## Abstract

Methicillin-resistant Staphylococcus aureus sequence type 72 (ST72) is prevalent in South Korea and has shown resistance to multiple antimicrobials. ST72 isolates display different levels of resistance to the antistaphylococcal lysostaphin. Draft genome sequencing of ST72 human isolates exhibiting lysostaphin resistance or susceptibility was performed to better understand the mechanism of lysostaphin resistance using subtractive genomics.

## ANNOUNCEMENT

Community-associated methicillin-resistant Staphylococcus aureus (MRSA) sequence type 72 (ST72) has emerged as a nosocomial infection that causes pneumonia, endocarditis, and skin and soft tissue infections (SSTIs) in South Korea (1, 2). Due to the multidrug resistance of community-associated ST72, we evaluated the efficacy of lysostaphin as an effective antistaphylococcal bacteriocin that specifically eradicates S. aureus by cleaving the pentaglycine bridge in the cell wall (3, 4).

**FIG 1:**
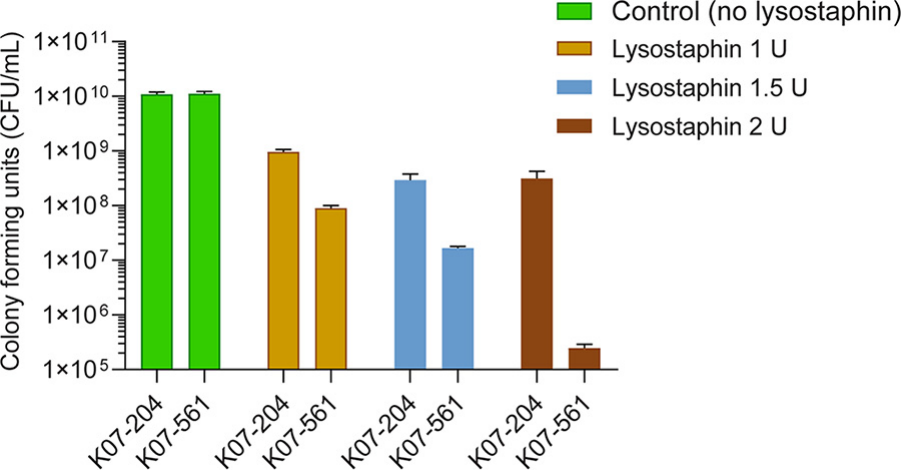
Responses of S. aureus ST72 strains to lysostaphin. Differential responses of K07-204 and K07-561 at various concentrations of lysostaphin are shown.

Human ST72 isolates (K07-561 and K07-204) used in the study were originally isolated by the Asia Pacific Foundation for Infectious Diseases, in South Korea (2). The different levels of susceptibility/resistance of K07-561 and K07-204 to lysostaphin were evaluated (4). The isolate K07-561 showed lysostaphin susceptibility, while K07-204 displayed an ∼1,000 times higher level of lysostaphin resistance upon treatment with 2 U of lysostaphin for 5 min (Fig. 1). These human ST72 isolates, K07-204 (lysostaphin resistant) and K07-561 (lysostaphin susceptible), represent an attractive model for elucidating the unknown mechanism of antibiotic and enzybiotic resistance. Therefore, we selected the isolates K07-204 and K07-561 as representative strains for genome sequencing to understand the genetic differences between the resistant and sensitive phenotypes.

A single colony was inoculated in 10 ml of tryptic soy broth (TSB) under orbital shaking culture conditions (200 rpm) at 37°C for 16 h. The cells were harvested by centrifugation. Genomic DNA from K07-204 and K07-561 was isolated by the phenol-chloroform extraction method (5). Briefly, the high-molecular-weight genomic DNA was sheared by using a Covaris S2 ultrasonicator system to obtain 350-bp DNA fragments. Libraries were prepared with the TruSeq Nano DNA sample preparation kit (Illumina, Inc., San Diego, CA, USA) by following the manufacturer’s protocol. Products were quantified using a Bioanalyzer 2100 system (Agilent, Santa Clara, CA, USA). Genomes were sequenced with a 150-bp paired-end Illumina NovaSeq 6000 platform. The quality control results were generated using FastQC software (v.0.10.1). Trimming of low-quality bases with a score below Q20 was performed using Sickle (v.1.3.3) (6), resulting in 21,289,096 and 28,904,426 surviving read pairs for K07-204 and K07-561, respectively. The genomes were assembled *de novo* using IDBA-UD (v.1.1.1) (7). The genome assembly of K07-204 and K07-561 yielded 66 and 58 contigs, respectively. The predicted lengths of the *de novo*-assembled genomes of K07-204 and K07-561 were found to be 2,310,534 bp and 2,149,433 bp, respectively. The GC contents of K07-204 and K07-561 were 32.92% and 32.88%, respectively. Contigs were annotated using the Prokaryotic Genome Annotation Pipeline (PGAP) (8). The K07-204 genome contained 2,168 coding genes, 40 tRNA sequences, and 1 rRNA sequence. K07-561 contained 2,040 coding genes and 21 tRNA sequences in its genome. The available genome sequences and their subtractive genomics in future studies will not only decode the novel genetic basis of the mechanisms of resistance to antibiotics and lysostaphin but also broaden the therapeutic interventions used against MRSA.

### Data availability.

The draft genome sequencing data for K07-204 and K07-561 are available in the GenBank database under the accession numbers JACSIU000000000.1 and JACORE000000000.1, respectively. Raw sequencing reads for K07-204 and K07-561 were deposited in the Sequence Read Archive (SRA) under BioProject/BioSample accession numbers PRJNA637991/SAMN15163378 and PRJNA637996/SAMN15163770, respectively.
